# Psychological Impact and Coping Strategies of Hispanic Parents of Children with Cancer: A Qualitative Study

**DOI:** 10.3390/ijerph20115928

**Published:** 2023-05-23

**Authors:** Carol Y. Ochoa-Dominguez, Kimberly A. Miller, Matthew P. Banegas, Daniel Sabater-Minarim, Randall Y. Chan

**Affiliations:** 1 Department of Radiation Medicine and Applied Sciences, University of California San Diego, La Jolla, CA 92037, USA; 2 Department of Population and Public Health Sciences, Keck School of Medicine, University of Southern California, Los Angeles, CA 90032, USA; 3 Center for Health Equity Education and Research, University of California San Diego, La Jolla, CA 92037, USA; 4 Department of Dermatology, Keck School of Medicine, University of Southern California, Los Angeles, CA 90033, USA; 5 Department of Biological Sciences, University of California San Diego, San Diego, CA 92161, USA; 6 Department of Pediatrics, Keck School of Medicine, University of Southern California, Los Angeles, CA 90033, USA

**Keywords:** parents, childhood cancer survivors, psychological impact, coping strategies, Hispanics

## Abstract

Throughout the cancer trajectory, parents of childhood cancer survivors (CCSs) may experience mental and social challenges requiring continual adaptation to cancer-induced stress. Using Lazarus and Folkman’s Transactional Model of Stress and Coping framework, this qualitative study aimed to describe Hispanic parents’ psychological health and explore their coping strategies. Purposive sampling was used to recruit 15 Hispanic caregivers from a safety-net hospital in Los Angeles County. To be eligible, participants had to be: the primary caregiver of a CCS who had completed active treatment, the primary caregiver or child self-identified as Hispanic, and proficient in English or Spanish. The interviews lasted approximately 60 min, were audio-recorded (in English and Spanish), and professionally transcribed. Data were analyzed following a thematic content analysis with deductive and inductive approaches on Dedoose. Participants described high levels of stress and fear when their child was diagnosed with cancer. They also shared experiencing symptoms of social anxiety, post-traumatic stress disorder, and depression. Participants’ coping strategies were encompassed by three major themes: problem-focused, emotion-focused, and avoidant coping strategies. Problem-focused coping strategies included self-efficacy, behavioral change, and social support. Emotion-focused coping strategies included religious practices and positive reframing. Avoidant coping strategies included denial and self-distraction. Despite the evident disparities in psychological health for Hispanic parents of CCSs, gaps remain in designing a culturally tailored program to help alleviate the caregiver burden. This study provides insights regarding coping strategies that Hispanic caregivers use to deal with the psychological impact of their child’s cancer diagnosis. Our findings also delve into the contextual and cultural factors that impact psychological adjustment.

## 1. Introduction

Childhood cancer is a life-altering event for both the child and their parents, with long-term impact on their psychosocial health. In light of increasing survival rates over time, there are more than 500,000 childhood cancer survivors in the United States [1]. Cancer remains a leading cause of mortality in the first two decades of life, however, trailing only behind trauma [2,3]. Treatment for childhood cancer has traditionally focused heavily on high-intensity therapies (e.g., submyeloablative chemotherapy, craniospinal irradiation, hematopoietic stem cell transplant) that trade short-term toxicities to achieve long-term cures [4]. Overwhelming bacterial infections, long hospital stays, intensive care support (e.g., mechanical ventilation), and extensive mucositis are among the most common, if not universal, occurrences that childhood cancer patients experience [5]. Additionally, while advancements in cancer treatments have resulted in an increasing number of childhood cancer survivors (CCSs), these individuals often experience a range of physical, emotional, and social challenges long after treatment [6,7]. 

Parents of CCSs play a critical role in providing medical, emotional, and practical support to their children throughout the cancer trajectory [8]. Caregiving can also be burdensome, however, and many caregivers of CCSs frequently report feelings of emotional distress, depression, and anxiety [9,10,11]. Caregiver burden is a complex and multidimensional concept that refers to the emotional, physical, and financial strain experienced by individuals as they care for a loved one with a chronic or life-threatening illness [12]. Parents of CCSs often are often required to provide medical assistance without previous training or outside support, while also continuing to fulfill their everyday activities [13]. Caregivers assist in giving domestic care, general care, and intimate care, which can lead to an overwhelming amount of pressure and feelings of burden [14]. Previous research has demonstrated that caregivers who experience high levels of burden are at increased risk of developing psychological distress, which can negatively impact their mental and physical health [15,16]. Most studies have focused on adult cancer survivors and spousal caregivers, yet little is understood about the experiences of caregivers of CCSs, particularly regarding their psychosocial health and coping strategies. 

The psychological adjustment of caregivers of CCSs is a dynamic process that involves adopting social behaviors and coping strategies to manage and overcome the psychosocial challenges associated with caregiving [17]. Lazarus and Folkman’s Transactional Model of Stress and Coping provides a framework for understanding the coping strategies employed by caregivers in response to stressors related to caregiving [18,19]. These stressors can stem from the damage already experienced through witnessing a loved one go through cancer treatment, the anticipation of cancer recurrence, and challenges that arise from providing intimate care [20]. An individual’s capacity to confront and adjust to these challenges depends on their adaptation level, as well as outside support, including familial, emotional, informational, or instrumental support [21,22]. 

Moreover, it is important to consider age-specific and cultural-specific beliefs and norms that may influence how an individual adapts to caregiving. Hispanic caregivers of CCSs may face unique challenges associated with their cultural background that may influence how they handle the demands of caregiving. For example, research has shown that Hispanic caregivers experience high levels of social isolation and lack of access to culturally appropriate support services, which can negatively impact their psychosocial health and well-being [23]. However, there is a lack of literature focusing on Hispanic caregivers of children with cancer. It is essential to understand the experiences of Hispanic caregivers of CCSs so that we can develop effective interventions to help reduce caregiver burden and improve psychosocial health in this distinct population. The objectives of this qualitative study were to collect in-depth information about the unique challenges and experiences of Hispanic caregivers of CCSs and, specifically, how this impacts their psychological health and the coping strategies. 

## 2. Methods

### 2.1. Study Design and Recruitment

We used semi structured in-depth interviews with individual caregivers to collect participants’ experiences with caregiving. One-on-one interviews were selected as the preferred method of data collection due to the nature of the research topic, i.e., wanting to understand the everyday lived experiences of Hispanic caregivers, who are an understudied population [24,25].

Ethics approval was obtained from the University of Southern California’s Institutional Review Board. Eligible participants were identified via chart review by R.Y.C. (medical oncologist), who then approached eligible participants while they waited for their CCS clinical visit with a brief description of the study. If the eligible participant expressed interest, then C.Y.O.D. (the principal investigator [P.I.]) was contacted via phone to discuss the study purpose, address any questions, and schedule the participant for one-on-one interview (previously described in greater detail) [7].

We used a purposive sampling method to recruit caregivers of childhood cancer survivors from the pediatric hematology–oncology clinic within the Los Angeles General Medical Center (LAGMC, previously the Los Angeles County + University of Southern California Medical Center). Los Angeles General Medical Center is a safety-net facility restricted to caring for low-income/no-income residents of Los Angeles County, defined as those who are eligible for benefits from the federal and state comanaged Medicaid program and/or Children’s Health Insurance Program—in California, adults are eligible if they earn 133% of the federal poverty level (FPL) or less, and children are eligible if families earn 261% of FPL or less. Caregivers were eligible for study inclusion if they were: (1) the primary caregiver of a CCS who had completed treatment, (2) either the primary caregiver or the CCS self-identified as Hispanic, and (3) fluent in English or Spanish. 

### 2.2. Interview Guide, Data Collection, and Study Sample

The interview guide was developed by C.Y.O.D., R.Y.C., and K.A.M. to explore multiple topics related to the caregiving experience based on a review of the literature and clinical observation of the target population. These questions were open-ended and contained various probes to gather a detailed description of caregivers’ experiences with the following topics: (1) barriers and facilitators, (2) psychosocial adjustment, (3) social support, and (4) communication. Specifically, questions focused on psychosocial adjustment included: (1) How have you been feeling since your child finished his/her cancer treatment?, (2) Can you describe how you think your mental health or well-being has been affected by your child’s cancer experience? (3) What are some things that make it easier for you to take care of your child’s health?, and (4) Could you tell me about some of the positive things you have encountered providing care and support for your child as a result of his/her condition?

Recruitment and data collection occurred in the summer of 2020, which was during the COVID-19 pandemic and stay-at-home advisory in California. For context, LAGMC did not halt in-person patient care, although many routine visits were rapidly transitioned to care via telephone and all in-person research was halted during the timespan in which this study was conducted. Consequently, after recruitment by the oncologist (which may have been via in-person visit for medical care or via telephone call), the P.I. contacted the participant by phone at their scheduled time and started with a brief overview of the study and asked the participant if they had any questions. Then, the P.I. proceeded to obtain verbal consent prior to starting the interview. 

Fifteen participants were interviewed for this study. The participants were composed of mostly mothers (*n* = 14, and one father) who were Spanish-speaking (*n* = 10), foreign-born (*n* = 11), and mostly married (*n* = 10, two single and three divorced/separated). The mean ages of parents and CCSs were 39 years (ranged: 23–58) and 7 years (ranged: 1–14 years), respectively. CCSs had been diagnosed with leukemia (*n* = 10), Hodgkin lymphoma (*n* = 2), sarcoma (*n* = 1), ovarian (*n* = 1), and unknown (*n*- = 1, parent was unable to recall the type of cancer). Specifically, for data collection, all CCSs had completed treatment for at least 6 months, and specifically the length of time since treatment completion ranged from <1 year (*n* = 4), 1–2 years (*n* = 2), to 2+ years (*n* = 9). All interviews were audio recorded, transcribed verbatim by a professional transcription service (in English or Spanish), and reviewed for accuracy by the P.I. Each interview lasted between 45 and 60 min. At the conclusion of the interview, all participants were mailed a $25 gift card. 

### 2.3. Data Analysis

To conserve the meaning of the interviews, they were transcribed and analyzed in the language in which they were conducted. Deidentified transcripts were uploaded into Dedoose, a qualitative data analysis software program, version 9.0.82 [26]. The approach used in this study was a thematic content analysis with deductive and inductive methods, whereby an initial codebook was created based on existing framework and literature (deducted), and refined based on the data (inductive) [27]. We used the Lazarus and Folkman’s Transactional Model of Stress and Coping framework and previous research to develop our initial codebook [18,19]. Then, two researchers (C.Y.O.D. and M.P.B.) read transcripts to identify additional concepts and themes from the interview data, which led to the codebook being revised. C.Y.O.D. then defined and provided examples for the final codebook and met with D.S.M. to discuss the final codebook before the formal coding took place. The first three transcripts were double-coded by C.Y.O.D. and D.S.M. to ensure consistency in coding. This involved meeting after coding each of those three transcripts to discuss the coding process. Then, the remaining twelve transcripts were coded separately by the two coders and analytical memos were written to enhance study rigor [28]. Finalized coded concepts and themes are *italicized* in our results described below.

## 3. Results

**Figure 1:**
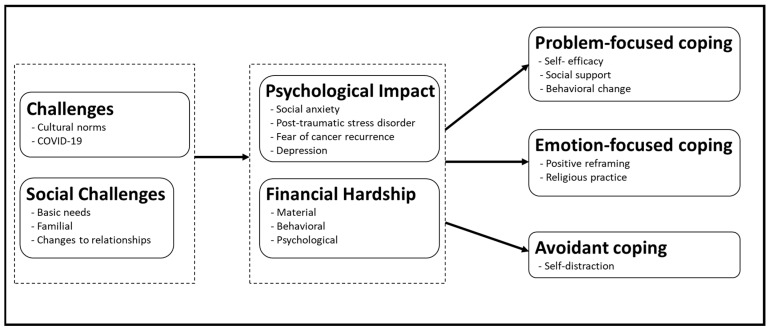
Conceptual model of contextual challenges, psychological impact, and coping strategies.

Throughout the interviews, caregivers described contextual challenges and the psychological impact of those challenges experienced during and after cancer therapy, and the coping strategies they developed because of and in response to those challenges and traumatic experiences. Figure 1 shows a graphic summary of our conceptual framework that links the impact as well as mitigation strategies (i.e., *stress and coping*, respectively) to their source.

### 3.1. Challenges

All the participants discussed experiencing various challenges before, during, and after their child’s diagnosis. Social challenges that they encountered included *childcare and family issues*, particularly if they had other children or were single parents. Others shared experiencing *changes to relationship*, such as family and friends distancing themselves from the caregiver, as well as parents’ not being able to be present for other family members due to the high needs of the CCS. For example, participant # 15 shared that a challenge she encountered was that her father became ill around the time the CCS was receiving cancer treatment, and that, because the child’s cancer treatment was so demanding, she was unable to see the father before he passed away. In the most extreme cases, changes in relationships included parents separating or divorcing after their child’s cancer diagnosis. Some participants had issues with meeting the *basic needs* of food, transportation, and housing, which made caregiving for their sick child extremely stressful. Participant # 8 described, “the [LAGMC] hospital is far away from us… so we took our daughter to closer clinic for therapy. We don’t have a car, and we have to pay for the bus fare for three, and we can’t… In fact, I have called the social worker to ask if they could help me pay my bills”.

Further, the majority of participants also believed that *COVID-19 created additional challenges* because it contributed to their feelings of isolation, lack of freedom, and heightened caregivers’ responsibilities and stress. Participant # 2 recalled, “It has been difficult to separate myself from [my parents] … They helped me with my children when I worked. Now with this… happening, I don’t know when they can come again”. Participant # 1 shared that, ever since the pandemic started, she has been constantly seeking God due to the fear of her son’s health. She explained, “I ask [God] to guard my son, because, as the doctor says, it would be very dangerous for him to get infected because he does not have defenses” and I tell my other kids “you have to be very careful because if one of us gets [covid] we can survive it, but if your brother gets it, he might not”. 

### 3.2. Psychological and Financial Impact

These challenges took a toll and impacted participants’ psychological and financial well-being. Participants described high levels of *stress, fear, and depression* when their child was diagnosed with cancer. Participants reported that “a sadness” came over them as they described cancer as an “ugly disease”. For example, participant # 10 recounted that when she received the diagnosis, “at first, I would just cry and cry… It made me sad… I was depressed…”. Another participant described that her son was calmer than her despite being the patient; when the diagnosis was delivered, she described feeling “like [her] world collapsed”. Further, when asked how they thought their emotional and mental health was impacted participant # 3, shared the following: “I think so, throughout the entire course of the [caregiving journey], and more when I had to take him to chemotherapy, I had a terrible urge to cry and cry”. 

During the time their children were receiving treatment, participants also shared experiencing *social anxiety and post-traumatic stress disorder* (PTSD) related to the delicacy of their child’s health. For example, participant # 7 recalled how traumatizing it was to see their child’s “hair fall out”. Specifically, when asked if the caregiving experience has affected her, she shared “Yes… it affected me a lot because I would see him lying in bed with a lot of ‘patches’ on his chest… I was more traumatized when I bathed him… and his hair fell out”. However, participant # 3, shared that the extended hospital stays were the most traumatizing part of the experience. She explained: “whenever I pass by the street Marengo or when I get on the freeway 5 coming from my work, it comes back, and I feel [how] I did when I was going to the [hospital]. When I went in the early morning… to the hospital… for urgent care… Because my biggest fear was losing my son… And I think that fear is still there”. Social anxiety was described by participant # 4 as the desire to keep her child “in a bubble” to keep from getting sick and to prevent long hospital stays. An example of this was shared by participant # 6, “I’m kind of overprotective… when he tries to roughhouse [with others], because I know that the chemotherapy, you know, weakens their bones and affects how they fuse back together, if he were to break a bone, which he has”.

Even after completion of their child’s cancer treatment, participants continued to experience a *fear of cancer recurrence;* as one participant described, they “[didn’t] want to put their guard down” and it was a difficult couple of weeks to transition to “normal” as they worried about everything the child does. Long after the focus of visits had shifted from disease surveillance to overall survivorship care, participants found themselves consumed with fear at the thought of upcoming annual visits to the oncologist. Participant # 4 stated: “But every time you know that you still have an appointment, or you have something, that’s when your nerves or your, you know, everything comes back”. Besides the fear at annual visits, participant # 3 also shared “I think, [I will] always have that fear, that fear that he will go through [cancer] again… That it will return”. Similar sentiments were described by participant # 12, who said “Sometimes, I do fear that it might return or with my other children”. 

Participants expressed a strong sense of financial hardship, as their caregiving responsibilities made it difficult to meet their financial responsibilities. In most cases, participants experienced *material hardships* resulting from underemployment or unemployment. For example, participant # 11 recalls “it was very difficult… because my husband was [also] going to the hospital… [so] he didn’t work much”. This was echoed by participant # 9, “the most difficult thing… was [paying] the rent because when my daughter [would] get sick I would call my husband and he would go to the hospital to be with [us]”.

Participants also described the need to give up employment to be their child’s primary full-time caregiver; those that continued employment described consistently missing work for medical visits and long hospitalizations. As participant # 4 reported “I did have to permanently stop working since the moment I found out, you know, what was his diagnosis. It was very hard at the beginning, you know, being a single mom and not working”. Some of the participants described receiving terminations from their employment due to ongoing work disruptions. For example, participant # 8 shared: “Well, I didn’t work. They fired [my husband] because we would take her to chemotherapy and he would miss work, so they fired [him]”.

Additionally, participants shared that they had competing living expenses. Participants described the increasing difficulties paying for their multiple basic necessities, including housing and food, while providing care to their child with cancer. For example, participant # 6 shared: “I was… alone. Being at the hospital, I had to deal with a lot with having to be kicked out from where I used to live, from finding a place to live, while having my son all in hospital at once. It was very hard to find someone to want to help or help with finding resources for myself or my son or my daughter”. 

Further, participants discussed *behavioral hardships*, including having to sell possessions or borrow money from family or friends to pay rent, bills, and basic necessities. As participant # 8 shared, “I called my neighbor… and I said… ‘can you help me and lend me five dollars’? To buy milk and tortillas… It hurts to remember that today… but I needed it so I swallowed my pride and [borrowed it]”. Finally, *psychological hardships* consisted of participants worrying and stressing about how they would pay for their bills. As participant # 3 explained, the stress of not having an adequate income: “I work 13 and 14 h a day, I don’t have vacations, I can’t rest… because I know that [my] check is not enough. The check is limited”. Further, participant # 9 reported feeling like it was “impossible… to [pay] for rent” because they were in and out of the hospital… “And [her husband] would stop working, sometimes, he worked two or three days a week”.

### 3.3. Coping Strategies

Participants’ coping strategies included three major themes: problem-focused, emotion-focused, and avoidant coping. The most common coping strategy was problem-focused, which referred to trying to solve/change the problem directly causing their distress. Emotion-focused coping focused on regulating the emotions and distress related to the child’s diagnosis. The least common coping strategy, avoidant, referred to ways in which participants tried to suppress their emotions to deal with their child’s diagnosis.

Problem-focused coping strategies were described in terms of *self-efficacy, behavioral change, and social support*. Participants described developing a sense of control over their caregiving responsibilities and their environment (*self-efficacy)* over the course of the cancer care and into survivorship. They learned to manage their child’s cancer treatment (e.g., symptom management), participate in assertive communication with the healthcare team, and access information independently, all of which promoted a sense of parental self-agency. For instance, participant # 7 shared that, once they established a daily routine, they were able to dedicate themself fully to the child while still satisfactorily completing household chores. Specifically, they shared: “When he slept, I did my chores, I cleaned the house, I washed. When he woke up, I would focus 100% on him”. One caregiver described her perspective after developing parental self-agency, “I think it made me stronger as a mom. Like because going through that, it was very hard” (participant # 15). 

Participants shared *behavioral changes* that included preparing healthier meals, using sunblock, and hygiene practices to help protect their child’s weakened immune system. These behavioral changes became long-term practices for caregivers as well, as participant # 12 shared, “I try to feed them better, put sunscreen… I think I have to be more aware of everything now”. Some participants talked about feeling well prepared to take the precautionary measures necessary to deal with COVID-19 because they were already accustomed to social distancing, sanitizing, and wearing masks when their child was receiving cancer treatment. Participant # 14 said “it’s basically the same routines, it’s only that now, we have to be extra careful… not having contact with other people. We don’t visit nobody”.

All participants shared that *social support* from family members, friends, healthcare team, and coworkers contributed to their ability to focus on caregiving and helped them feel like they were not alone. One caregiver shared “My mom was my biggest supporter. She helped me on everything: financially, physically, she was there to support me” (participant # 14). Another caregiver shared, “it was difficult for me, but at the same time not so much because we had a lot of help. Here among the whole family, as we are many, they helped a lot” (participant # 13). Some participants shared that their family also provided them with monetary support, as they were unable to work. In one extreme case, the caregiver describes experiencing homelessness.

“During the first month of him being in the hospital, when he was first diagnosed, they kicked me out. So, I had nowhere to go. My aunt offered me a room in her place… And that’s where like a lot of the whole supports system comes into play. I didn’t really have much. The one to help me and see everything first hand, from how I dealt with things and how it was affecting me was my aunt”.

(participant # 6)

Emotion-focused coping strategies included *religious practices and positive reframing*. About half of the participants shared how their *religious practice* helped them emotionally deal with their child’s diagnosis. This usually involved the mention of God or a higher being and practices such as church attendance, prayer, reading of the Bible, and religious beliefs centered on accepting the illness and redirecting their attention to God to save their child. Through their faith and believe in God, participants were able to regulate their emotions and stay calm as their child went through cancer treatment, and that their child would not relapse. Besides the direct emotional support their faith provided, one participant shared that practicing her religion meant “being stronger, keep moving forward, and believing more in [God] and being more grateful. And valuing more what we have, to value life more, to value the moments… because we don’t know how tomorrow will be” (participant # 3). Another caregiver shared, “God helped me; he made me strong for my daughter to not see me defeated. To be ready to help my daughter. And thanks be to God everything has gone well” (participant # 13).

The majority of participants described the notion of *positive reframing* as focusing on positive thoughts, focusing on encouraging words (e.g., going to get cured, will get better) and increasing exposure to positive people, and reducing fixation on anxiety and sadness when such emotions arose. Participants described incidences such as a family member encouraging the participant to stay positive when anxious feelings would arise. Additionally, in some cases, the CCS was also able to help the participant by trying to cheer them up. For example, participant # 7 shared that their child would find ways to make them laugh, so they wouldn’t be sad when the child’s hair was falling out. Some participants shared the experience of reframing thoughts during follow-up visits to prevent overwhelming fear. Other participants shared that the cancer therapy experience ultimately improved their relationship with child, stating they “[were] closer and talked more” (participant # 10). Another participant shared that caring for the CCS made them a stronger parent, and they felt like “any challenge is possible” (participant # 14).

Avoidant coping strategies included *denial of and self-distraction* away from thoughts and feelings associated with their child’s cancer. Participants described the need to use these strategies primarily in front of their children. For example, participants shared the need to “present themselves as strong”, keep themselves from “crying” and “venting” to not demonstrate weakness. Among the four participants that described using avoidant coping, the most common strategy described was self-withdrawal. Another strategy shared by a couple of participants was to deliberately avoid sharing experiences with others. Participant # 3 explained that they were always serious because they “felt that if [the participant] spoke to someone… or someone asked [the participant] something, [the participant] was not going to be hold back and was going to cry”. 

## 4. Discussion

Findings from this study highlight the unique challenges, psychological impact, and coping strategies of Hispanic parents of CCSs who received care from a safety-net clinic in Los Angeles County. We found that all participants reported using problem-focused coping strategies and the majority (14 out of 15) also used emotion-focused coping strategies, though few reported using avoidant coping strategies.

Within this study, caregivers reported many challenges, including familial, cultural norms, meeting basic needs, and the impact of COVID-19. Previous research has shown that Hispanic caregivers of adult cancer survivors experience personal, financial, and structural challenges, including high financial burden and low earnings, challenging employment environment, and juggling multiple tasks of caregiving [29,30]. While we found that participants in our study experienced similar challenges, we also explored the psychological impact on caregivers and their coping strategies in depth. The results of our work show that parents struggle with seeing their child undergo cancer treatment and moving forward “back to normalcy” after their child has completed treatment, contributing to their prolonged stress and trauma.

Notably, the psychological impact and coping strategies that the caregivers experienced were similar to those previously reported by childhood and adolescent and young adult (AYA) cancer survivors [6,11,21]. Specifically, it is well documented that CCSs and AYA survivors are at increased risk for anxiety, depression, and post-traumatic stress symptoms. Prior research has shown that all forms of social support (including the sharing of cancer information), positive reframing, religious practice, and avoidant practices are coping strategies CCSs and AYA survivors utilize [11,31,32,33,34,35]. While we acknowledge that these coping strategies are similar to our findings, more research is needed to further explore the factors that contribute to the development and use of these coping strategies, particularly for younger versus older patients and their caregivers. 

Further, in line with previous research, we found that Hispanic parents of CCSs experience fear of cancer recurrence [36,37]. It is important to address the fear of cancer recurrence and support parents coping strategies because Hispanic cancer patients and caregivers may experience higher levels of the already universal worry about recurrence than non-Hispanic whites, which can persist long after the medical team has shifted care focus away from disease surveillance and into survivorship [38,39]. Particular attention to the fear of recurrence is needed as the care of CCSs is shifted from the oncologist to the primary care physicians as well, the latter whom provide the bulk of survivorship care [40].

Moreover, the use of the Transactional Model of Stress and Coping as a theoretical framework enabled the identification of coping strategies parents use to combat these emotions. Our findings suggest that a potential intervention for parents may be warranted around the time of their child’s follow-up appointments. For example, a phone-based intervention culturally specific to Hispanic parents that provides a space for them to discuss concerns regarding the upcoming doctor visit and may help ease parents’ emotions by ideally reframing their thoughts. 

While less commonly used, we did see that some parents used avoidant coping strategies to not focus on their child’s cancer diagnosis and the related emotions. This finding aligns with a previous qualitative study among middle-aged and older Hispanic caregivers of people with Alzheimer’s disease or related dementia, in which caregivers described ignoring the issues, distracting themselves, and keeping busy to suppress their emotions [41]. Our study builds on previous research by discussing the reason why these Hispanic parents use this coping strategy, which was to “present themselves as strong”, keep themselves from “crying”, and “venting” to not demonstrate weakness. While this strategy may be effective in the moment, as it affords parents the opportunity to keep tackling their caregiving responsibilities, ultimately it may be maladaptive, as it has the potential to exacerbate the psychological impact of the experience. For example, in a study of 139 parents of childhood cancer survivors, it was found that Hispanic parents reported significantly more post-traumatic stress and depressive symptoms than non-Hispanic parents [42]. Ultimately, maladaptive strategies need to be screened for and addressed as part of cancer and survivorship care, as they have been shown to actually increase caregiver burden over time [43].

This study also has some limitations to consider. First, the sample was a relatively small purposive sample of Hispanic parents from Los Angeles County and may not reflect the larger U.S. Latino population. Second, interviews were conducted during the beginning of the COVID-19 pandemic, which resulted in all in-depth interviews being conducted by phone, which may have limited participant responses. However, previous studies suggest that phone interviews are a commonly used method for data collection and are highly suitable [44,45]. Our experience was that recruitment and data collection was a quick process and participants did not express concerns with the telephone interview. Further, our interviews lasted between 45 and 60 min, and our data were rich in details, as participants felt comfortable sharing their stories with us. Lastly, the data collected may be susceptible to recall bias. Future studies should consider recruiting from multiple hospitals, different regions of the country with diverse Latino heritage groups, and further exploring the topics discussed by our interviewees. 

## 5. Conclusions

This study provides insights regarding coping strategies that Hispanic caregivers use to deal with the psychological impact of their child’s cancer diagnosis. Our findings also delve into the contextual factors that impact psychological adjustment. Despite the evident psychological challenges that Hispanic parents of CCSs experience, gaps remain in the availability of culturally tailored programs. Future interventions are needed to target the distinct experiences of Hispanic caregivers of CCSs to alleviate caregiver burden and distress.

## Data Availability

Not applicable.
